# Treatment Effectiveness of Clear Aligners in Correcting Complicated and Severe Malocclusion Cases Compared to Fixed Orthodontic Appliances: A Systematic Review

**DOI:** 10.7759/cureus.38311

**Published:** 2023-04-29

**Authors:** Samer T Jaber, Mohammad Y Hajeer, Kinda Sultan

**Affiliations:** 1 Department of Orthodontics, University of Damascus, Damascus, SYR

**Keywords:** crowding of teeth, clinical effectiveness, premolar extraction, systematic review, effectiveness, invisalign, clear aligners, orthodontic

## Abstract

This systematic review aimed to critically assess the available evidence regarding the effectiveness and efficiency of clear aligners in the comprehensive treatment of complex cases accompanied by premolars extraction. An electronic literature search by two reviewers was independently done on 27 February 2023 in the following databases without time and language limitations: Pubmed®, Scoups®, Google Scholar, Cochrane Library database, Web of Science™, and Proquest Database Open. Randomized controlled trials (RCTs) of any type, non-randomized clinical trials (CCT), cohort studies, and prospective, retrospective, and cross-sectional studies were reviewed. The risk of bias in included studies was assessed using the Risk of Bias (RoB 2.0) tool for randomized trials and the Risk of Bias in Non-randomized Studies (ROBINS-I) tool for non-randomized studies. After carefully searching the literature, six trials were included in this systematic review, three RCTs, two retrospective cohort studies, and one CCT. Two hundred eighty-three patients were included (186 females, 97 males). Three studies found that there were no differences between the clear aligners and fixed appliances when evaluations were done using the American Board of Orthodontists Objective Grading System (ABO-OGS) or the Peer Assessment Rating (PAR) index. Two studies found that there were some differences between predicted and achieved tooth movements when clear aligners were used in premolars extraction cases. Based on the included studies, the duration of treatment was shorter with fixed appliances than the clear aligners when applied to orthodontic extraction cases. Both clear aligners and fixed appliances were found effective in the orthodontic treatment of premolar extraction-based cases. Fixed appliances have the advantage of achieving better buccolingual inclination and occlusal contacts in a shorter treatment duration. Treatment with clear aligners might be associated with differences between predicted and achieved tooth movements. Therefore, the characteristics of these techniques should be considered when making a treatment decision.

## Introduction and background

Malocclusion is often considered to have a negative impact on self-esteem and physical, social, and psychological well-being [1]. Therefore, adults seek orthodontics treatment to enhance their smile, occlusion, psychological well-being, and quality of life [2,3]. However, some of them resist proceeding with the treatment due to the long treatment duration, discomfort, cost, and the unaesthetic appearance accompanied by the conventional buccal fixed appliances [4]. With the advances in the orthodontic field, invisible techniques have been introduced to patients offering them an aesthetic orthodontic treatment. The lingual appliances provided an aesthetic advantage over the buccal ones, but their use was limited due to the multiple difficulties associated with them [5-8]. In 1997, Invisalign® (Align Technology, San Jose, California) was introduced, rendering Kesling's ideas a feasible orthodontic treatment option [9]. Clear aligners are a series of removable plastic appliances that are used for moving teeth [10]. They have become more popular due to the rapid development of digital three-dimensional (3D) technologies and dental materials [11,12].

With the advent of Invisalign®, the use of aligners was indicated for treating mild to moderate degrees of crowding, mild to moderate spaces, correction of dental flaring and tipping, and for relapsed cases treated previously using fixed appliances [13]. Over time, the range of the treated cases with clear aligners has expanded from the treatment of simple orthodontic cases to more complex cases such as treatment of anterior open bite [14], bimaxillary protrusion [15], excessive facial height, and severe dental crowding [16]. Treatment plans for these cases are often accompanied by premolars extraction, and this can present several challenges during treatment, such as posterior anchorage control [17] and preserving the stability of the closed spaces after treatment by ensuring root parallelism of the teeth adjacent to the extraction sites [18].

In the last decade, several reviews have emerged to evaluate the efficiency, effectiveness, and accuracy of aligners in orthodontic treatments [19-21]. However, non of these reviews explicitly studied the scientific evidence that supports the effectiveness and efficiency of clear aligners in the orthodontic treatment of complex extraction-based cases and their ability to achieve proper occlusion at the end of the treatment. Therefore, this systematic review aimed to critically assess the available evidence regarding the effectiveness and efficiency of clear aligners in the comprehensive treatment of complex cases accompanied by premolars extraction. The focused review question was: "Are clear aligners effective in treating complex extraction-based cases, and are they better than fixed appliances?".

## Review

Materials & methods

Protocol and Registration

Before writing up the final protocol of this systematic review, a PubMed scoping search was performed to verify the existence of similar systematic reviews and to explore potentially eligible articles. No systematic review was found in the literature evaluating clear aligners' effectiveness in treating complex extraction-based cases. During the first stage of the review, registration was done in the International Prospective Register of Systematic Reviews (PROSPERO; CRD42022367851). This review was done according to the Cochrane Handbook [22] and the Preferred Reporting Items for Systematic Reviews and Meta‐Analyses (PRISMA) statement [23].

Eligibility Criteria

Inclusion and exclusion criteria were established according to the Participants, Intervention, Comparisons, Outcomes, and Study design (PICOS) framework, which was developed based on the focused review question as follows; Participants: healthy human patients of any age, both genders, of all ethnic groups who have complicated and severe malocclusion, and their treatment involved extraction of premolars (four or two premolars). Intervention: orthodontic treatment using clear aligners. Comparisons: in the case of comparative studies, the comparisons were made with fixed appliances. Outcomes: the primary outcome was the comprehensive orthodontic treatment outcome evaluated using objective and reliable assessment methods like the American Board of Orthodontics objective grading system (ABO-OGS), Peer Assessment Rating (PAR) index, and evaluation of dental movements using analysis or superimposition of pre-and posttreatment records. The secondary outcome was the treatment duration. Study design: Randomized controlled trials (RCTs) of any type, non-randomized clinical trials (CCTs), and cohort studies. Prospective, retrospective, and cross-sectional studies were reviewed. No limitations concerning language or publication year were applied.

Search Strategy

An electronic literature search by two reviewers (Samer T. Jaber (STJ) and Mohammad Y. Hajeer (MYH)) was independently done on 27 February 2023 in the following databases without time and language limitations: Pubmed®, Scoups®, Google Scholar, Cochrane Library database, Web of Science™, and Proquest Database Open (to identify dissertations and theses). Keywords and the details of the search strategy used are provided in Table 1. The reference lists of selected papers and relevant reviews were screened for possible related studies that the electronic web-based search may not have discovered.

**Table 1:** The electronic search strategy

Database	Search strategy
CENTRAL (The Cochrane Library)	#1 orthodontic* OR "clear aligner" OR "orthodontic appliances'' OR "orthodontic clear aligner" OR "Invisalign" OR "aligners'' OR ''transparent aligners'' OR '' removable orthodontic appliances'' OR ''orthodontic treatment" OR "orthodontic Therapy" #2 effect* OR effic* OR achieve OR predict OR outcome* OR advent* OR duration OR time. #3 extraction OR four premolars extraction OR two premolars extraction. #4 malocclusion OR crowding OR bimaxillary protrusion*. #5 #3 OR #4 #6 #1 AND #2 AND #5
PubMed	#1 orthodontic* OR "clear aligner" OR "orthodontic appliances'' OR "orthodontic clear aligner" OR "Invisalign" OR "aligners'' OR ''transparent aligners'' OR '' removable orthodontic appliances'' OR ''orthodontic treatment" OR "orthodontic Therapy" #2 effect* OR effic* OR achieve OR predict OR outcome* OR advent* OR duration OR time. #3 extraction OR four premolars extraction OR two premolars extraction. #4 malocclusion OR crowding OR bimaxillary protrusion*. #5 #3 OR #4 #6 #1 AND #2 AND #5
EMBASE	#1 orthodontic* OR "clear aligner" OR "orthodontic appliances'' OR "orthodontic clear aligner" OR "Invisalign" OR "aligners'' OR ''transparent aligners'' OR '' removable orthodontic appliances'' OR ''orthodontic Treatment" OR "orthodontic Therapy" #2 effect* OR effic* OR achieve OR predict OR outcome* OR advent* OR duration OR time. #3 extraction OR four premolars extraction OR two premolars extraction. #4 malocclusion OR crowding OR bimaxillary protrusion*. #5 #3 OR #4 #6 #1 AND #2 AND #5
Google Scholar	#1 (clear aligner OR Invisalign) AND (effect OR effici) #2 orthodontic AND (clear aligner OR Invisalign) AND (severe crowding OR bimaxillary protrusion) #3 (clear aligner OR Invisalign) AND (extraction OR four premolars extraction OR two premolars extraction) #4 (clear aligner OR Invisalign) AND (predict OR achive OR actual)
Scopus	#1 TITLE-ABS-KEY (orthodontic* OR "Invisalign" OR "clear aligners" OR "orthodontic appliances"). #2 TITLE-ABS-KEY (effect* OR effic* OR predict* OR achieve* OR outcome) #3TITLEABS- KEY ("clear aligner" OR "Invisalign" OR "tranparent aligners") AND (extracion* OR four premolars extraction* OR two premolars extraction*) #4 TITLE-ABS-KEY ("clear aligner" OR "Invisalign") AND (severe crowding* OR bimaxillary protrusion* OR malocclusion*) #5 #1 AND #2 #6 #3 AND #5 #7 #4 AND #5
Web of Science	TS = (orthodontic applianc* OR orthodontic brack*) AND TS = (Invisalign OR clear align* OR invisible applianc* OR orthodontic align* OR removable thermoplastic applianc*) AND TS = (orthodontic* OR malocclusion OR tooth crowding OR orthodontic treatment OR orthodontic patient*)
Proquest Database Open	#1 (clear aligner OR Invisalign) AND (effect OR effici) #2 orthodontic AND (clear aligner OR Invisalign) AND (severe crowding OR bimaxillary protrusion) #3 (clear aligner OR Invisalign) AND (extraction OR four premolars extraction OR two premolars extraction) #4 (clear aligner OR Invisalign) AND (predict OR achive OR actual)

Study Selection and Data Extraction

Two reviewers (STJ & MYH) independently assessed the articles for suitability according to the inclusion criteria; in case of disagreement, the third reviewer (Kinda Sultan (KS)) was asked to decide and resolve it. At first, the titles and abstracts of the articles were screened during the search using the inclusion criteria by the two reviewers. After that, the same two reviewers evaluated the full text of the screened articles that might be included in the review. Any article that did not fulfill one or more of the inclusion criteria was discarded from the review. Finally, the following data were extracted from each of the included articles using piloted and predefined extraction sheets: general information (authors name, publication date, journal, country), study design, participants' characteristics (size, age, eligibility criteria), intervention, teeth extracted, treatment duration, assessment methods, and outcomes.

Risk of Bias of Individual Studies

The risk of bias in the included studies was assessed according to Cochrane guidelines with the Risk of Bias (RoB 2.0) tool for randomized trials [24] and the Risk of Bias in Non-randomized Studies of Interventions (ROBINS-I) tool for non-randomized studies [25]. Assessment of the risk of bias within individual trials was likewise performed independently by the authors (STJ & MYH). The judgments of both authors were compared. In case of disagreement and could not reach a consensus by discussion, a third reviewer (KS) was asked to decide. For randomized studies, the following five domains were judged as having a high risk of bias, low risk of bias, and some concerns: randomization process, deviations from intended interventions, missing outcome data, measurement of the outcome, and selection of the reported result. After that, the overall risk-of-bias judgment was reached according to the following criteria: low risk of bias if all the domains were at low risk of bias, some concerns if there were some concerns in at least one domain without any high risk of bias for any other domain, and high risk of bias if one or more fields were assessed as being at high risk of bias or if there were some concerns for multiple domains in a way that substantially lowers confidence in the result. For the evaluation of the non-randomized clinical trials with the ROBINS-I tool, the evaluated criteria were divided into pre-intervention, intervention, and post-intervention categories. The risk of bias was individually analyzed for each study and classified as low, moderate, serious, critical, and no information.

Results

Literature Flow

**Figure 1:**
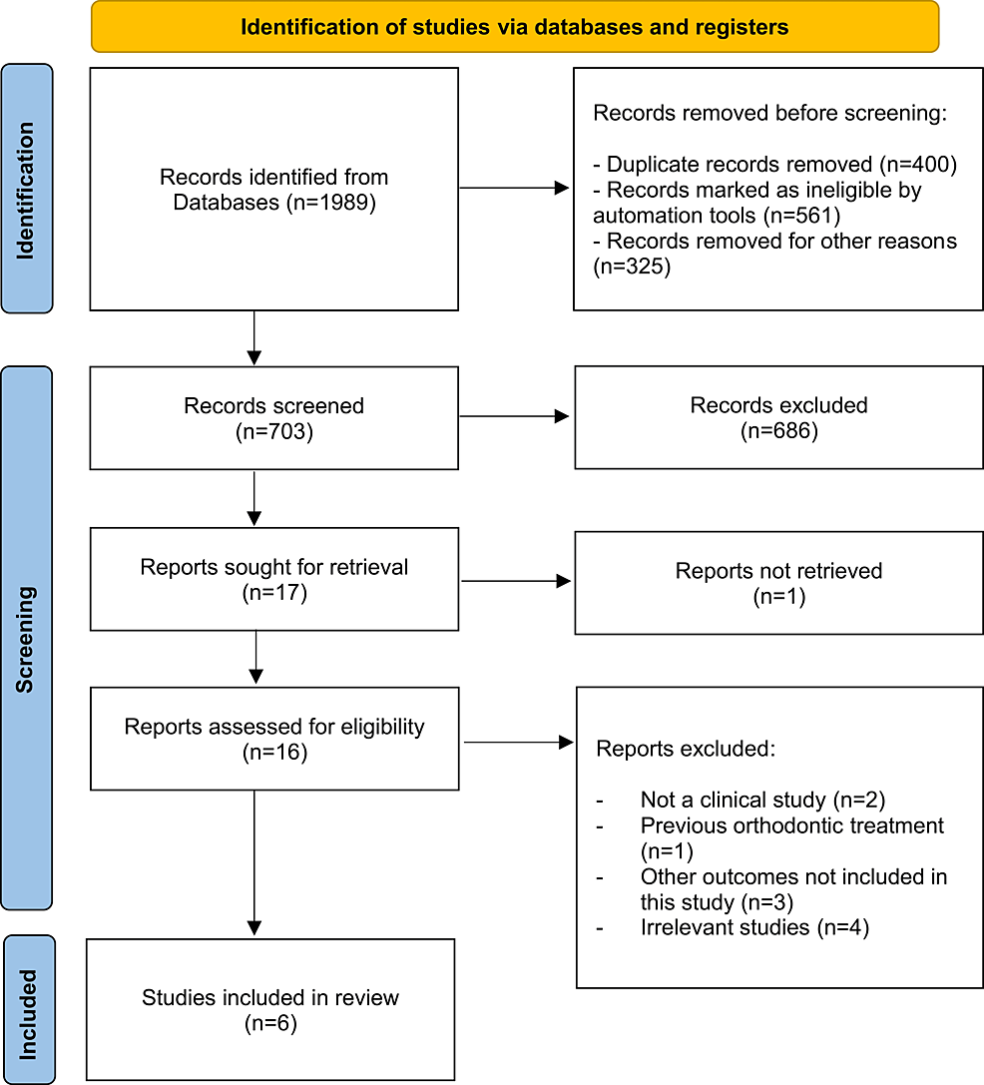
The Preferred Reporting Items for Systematic Reviews and Meta‐Analyses (PRISMA) flow diagram of the reviewing process.

A total of 1989 references were identified from an electronic databases search. One thousand two hundred eighty-six citations were removed because of duplication, ineligibility by automation tools, and other reasons. The titles and abstracts of 705 records were screened carefully for eligibility, and then all records which were not fulfilling the inclusion criteria were eliminated. As a result, 16 potentially related records were left for full-text assessment. Ten studies did not meet the inclusion criteria and were excluded from the review. Subsequently, six trials were included in this systematic review. Figure 1 illustrates the Preferred Reporting Items for Systematic Reviews and Meta‐Analyses (PRISMA) flow diagram of the reviewing process.

Characteristics of the Retrieved Studies

Characteristics of the six studies included in this review are given in Table 2. Out of the six included studies in this review, there were three RCTs [26-28], two retrospective cohort studies [29,30], and one CCT [16]. Three studies were from China [27,29,30]; three were from the USA [26], Italy [16], and Syria [28]. All of the studies included males and females. The total participants involved in the included studies were 283 patients (186 females, 97 males); 177 of them were treated with clear aligners, while the rest of them (106 patients) were treated with traditional buccal fixed appliances. The average age of the patients ranged from 19.4 to 32.8 years. Only one study did not provide information about the age range [16]. All the included studies used clear aligners provided by Invisalign® except for the Jaber et al. study, which used in-house clear aligners. Three of these studies had control groups treated with traditional fixed appliances [16,27,28]. Whereas the other three studies were not comparative, i.e., there weren't control groups included in these studies [26,29,30]. All of the studies involved extraction-based treatment; four of them included upper and lower first premolars extraction [16,27,28,30] and one with only upper first premolars [29]. The last study included cases with at least one premolar extraction [26]. Evaluation of clear aligners' effectiveness was done using either the occlusal indices or tooth movement measurements on superimposed digital dental models using a three-dimensional coordinate system generated for this purpose. Li et al. used the ABO-OGS to evaluate the cases after appliance removal, whereas Gaffuri et al. used the same ABO grading system and compared its components before and after treatment. Little's irregularity index (LII) and PAR index were used in Jaber et al. study, whereas analysis and measurements of case records, including dental casts and panoramic radiographs, were used in Baldwin et al. study. In contrast, Dai et al. used tooth movement measurements after superimposing actual and virtual dental models as their evaluation method in their two studies.

**Table 2:** Characteristics of the included studies in this systematic review RCT - randomized controlled trial, F - female, M - male, pts -  patients, ABO-OGS - American Board of Orthodontics - Objective Grading System, LII - Little's irregularity index, PAR - Peer Assessment Rating

Author (year)	Country	Study design	Population			Intervention	Treatment duration	Outcomes assessed	Assessment methods
			Total	Mean age	Inclusion criteria				
Baldwin et al. (2008) [26]	USA	RCT	24 pts (18 F, 6 M)	32.8 (42.7 M, 29.4 F)	Age ≥ 18 years, ability to attend weekly appointments and to pay for services, requirement for regular dental and periodontal maintenance program in case of caries or periodontal disease	Invisalign^®^ clear aligners	Aligners: 16.9 months, control: 23.2 months	Tooth tipping around extraction spaces	Analysis of dental casts and panoramic radiographs
Li et al. (2015) [27]	China	RCT	152 pts, control: 76 (45 F, 27 M), aligners: 76 (45 F, 27 M)	Control: 32.2 ± (8.3), aligners: 35.2 ± (7.3)	Extraction patients. Class I malocclusion; severity in complexity with discrepancy index score of 25, indicating high complexity	Control: Fixed appliances, aligners: Invisalign^®^ clear aligners	Aligners: 31.5 months, control: 22 months	ABO-OGS score system	Analysis of pre-and posttreatment records (study casts and radiographs)
Dai et al. (2019) [29]	China	Retrospective cohort study	30 pts (26 F, 4 M)	19.4 ± (6.3)	Patients who had been treated using clear aligners with extraction of two maxillary first premolars, and their first series of aligners finished with no midcourse correction	Invisalign^®^ clear aligners	22.3±(4.6) months for the first series of aligners	Differences between predicted and achieved movement for maxillary first molar and central incisors Effects of age, initial crowding, and attachments on molar anchorage control	Superimposition of actual pre-and posttreatment models with virtual pre-and posttreatment using Rapidform 2007 software
Gaffuri et al. (2020) 16]	Italy	CCT	24 pts (13 F, 11 M), control: 12, aligners: 12	-	Permanent dentition patients with severe crowding or bimaxillary malocclusion	Control: MBT fixed appliances, aligners: G6 Invisalign^®^ clear aligners	Aligners: 2.1 years plus maximum of 7 months for refinements, control: 2 years	ABO-OGS score system ABO standard cephalometric analysis	Pre- and posttreatment records (digital models and panoramic radiographs) were analyzed with AudaxCeph Advantage software
Dai et al. (2021) [30]	China	Retrospective cohort study	17 pts (15 F, 2 M)	25.4 ± (5.0)	Patients who had been treated using clear aligners with extraction of four first premolars, and their first series of aligners finished with no midcourse correction	Invisalign^®^ clear aligners	21.0±(4.2) months for the first series of aligners	Comparing the predicted and achieved crown movements of maxillary and mandibular first molars, canines, and central incisors	Superimposition of the pretreatment, predicted posttreatment and actual posttreatment maxillary and mandibular dental models.
Jaber et al. (2022) [28]	Syria	RCT	36 pts (24 F, 12 M)	21.30 ± (2.29)	Age 18-25 years Class I malocclusion with severe crowding, which requires the extraction of the first premolars	Control: Fixed appliances Aligners: In-house clear aligners	Aligners: 23.27 ± (5.82), control: 26.20 ± (5.27)	LII, PAR index, treatment duration	Analysis of pre-and posttreatment study casts

Risk of Bias in the Included Studies

**Figure 2:**
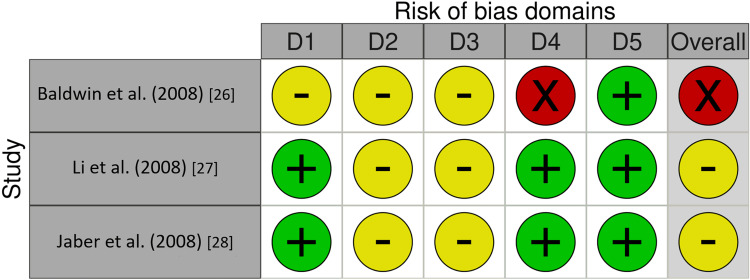
Risk of bias summary: the review authors' judgments about each item of the risk of bias for the randomized controlled trial studies Red circle - high risk of bias; yellow circle - some concerns; green circle - low risk of bias Domains: D1: Bias arising from the randomization process; D2: Bias due to deviations from the intended intervention; D3: Bias due to missing outcome data; D4: Bias in measurement of the outcome; D5: Bias in the selection of the reported result.

**Figure 3:**
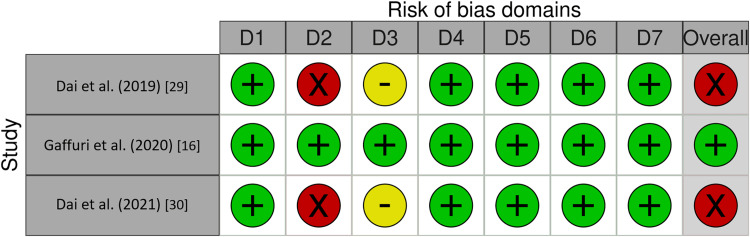
Risk of bias summary: the review authors' judgments about each item's risk of bias for the non-randomized controlled trials Red circle - serious risk of bias; yellow circle - moderate risk of bias; green circle - low risk of bias. Domains: D1: Bias due to confounding; D2: Bias due to selection of participants; D3: Bias in the classification of interventions; D4: Bias due to deviations from intended interventions; D5: Bias due to missing data; D6: Bias in measurement of outcomes; D7: Bias in selection of the reported result.

**Figure 4:**
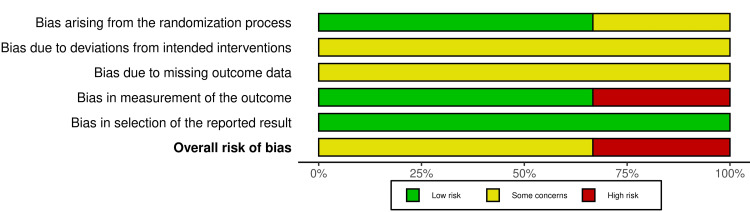
Risk of bias graph for the randomized controlled trials: the review authors' judgments about each item's risk of bias, presented as percentages across all the studies included

**Figure 5:**
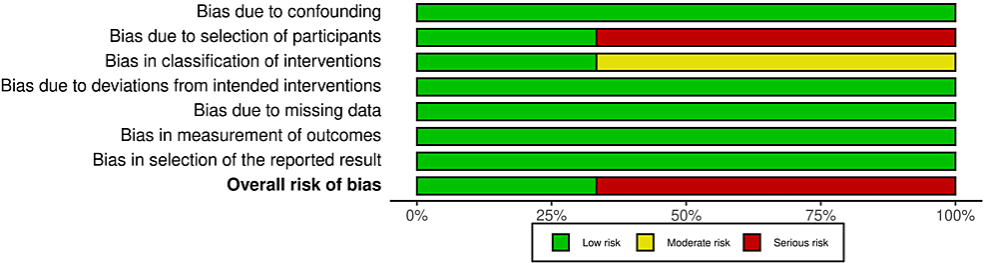
Risk of bias graph for the non-randomized controlled trials: the review authors' judgments about each item's risk of bias, presented as percentages across all the studies included

The summary of the risk of bias in the included studies is shown in Figures 2-5. For the RCTs, one was at high risk of bias [26], whereas the other had some concerns [27,28]. Blinding was the most problematic field in both studies. For the other three non-RCT included studies, two were at serious risk of bias [29,30], and one was at low risk [16]. Those with serious risks had severe issues with the selection of participants in the study and also had a moderate bias in the classification of interventions. More details about the risk of bias assessment and the supporting reasons for each assessment are given in Tables 3 and 4.

**Table 3:** Risk of bias of the included randomized controlled trials

Study	Domains						
	Bias arising from the randomization process	Bias due to deviations from intended interventions		Bias due to missing outcome data	Bias in the measurement of the outcome	Bias in the selection of the reported results	Overall bias
		Effect of assignment to interventions	Effect of adhering to interventions				
Baldwin et al. (2008)[26]	Some concerns: Randomization schedule is based on a list of random numbers performed by a calibrated investigator unaware of the treatment plan.	Some concerns: Blinding cannot be performed. There is ''no information'' on whether any deviations arose because of the trial context.	Some concerns: Blinding cannot be performed, and ''no information'' on whether the important non-protocol interventions were balanced across intervention groups.	Some concerns: Missing outcome data balanced in numbers across intervention groups, with similar reasons for missing data across groups.	High risk: The method of measuring the outcome was not appropriate. The outcome assessors were the principal investigator (who was not blinded for the assignment of each intervention) and the other two dentists who were blinded.	Low risk: The assessed numerical result has not probably been selected based on the results from multiple eligible outcome measurements within the outcome domain and analysis of the data. The eligible reported results for the outcome corresponded to the intended outcome measurements.	High risk: The study is judged to raise high risk because one of the domains got this result
Li et al. (2015)[27]	Low risk: Randomization using a computer-generated sequence performed by Statistical Analysis System. Randomization was concealed using opaque envelopes by two clinicians not involved in the study	Some concerns: Blinding cannot be performed. There is ''no information'' on whether any deviations arose because of the trial context.	Some concerns: Blinding cannot be performed, and ''no information'' on whether the important non-protocol interventions were balanced across intervention groups.	Low risk: No dropouts were reported	Low risk: The method of measuring the outcome was appropriate. The outcome assessor was blind for the assignment of each intervention.	Low risk: The assessed numerical result has not probably been selected based on the results from multiple eligible outcome measurements within the outcome domain and analysis of the data. The eligible reported results for the outcome corresponded to the intended outcome measurements.	Some concerns: The study is judged to raise some concerns because one of the domains got this result
Jaber et al. (2022)[28]	Low risk: Randomization using a computer-generated sequence performed by Statistical Analysis System. Randomization was concealed using opaque envelopes by two clinicians not involved in the study	Some concerns: Blinding cannot be performed. There is ''no information'' on whether any deviations arose because of the trial context.	Somxxxe concerns: Blinding cannot be performed, and ''no information'' on whether the important non-protocol interventions were balanced across intervention groups.	Low risk: No dropouts were reported	Low risk: The method of measuring the outcome was appropriate. The outcome assessor was blind for the assignment of each intervention.	Low risk: The assessed numerical result has not probably been selected based on the results from multiple eligible outcome measurements within the outcome domain and analysis of the data. The eligible reported results for the outcome corresponded to the intended outcome measurements.	Some concerns: The study is judged to raise some concerns because one of the domains got this result

**Table 4:** Risk of bias of the included non-randomized controlled trials

Study	Domains							
	Pre-intervention		Intervention	Post-intervention				Overall bias
	Bias due to confounding	Bias in selection of participants into the study	Bias in classification on interventions	Bias due to deviations from intended interventions	Bias due to missing data	Bias in the measurement of the outcome	Bias in selection of reported results	
Dai et al. (2019) [29]	Low: No confounding is expected.	Serious: Selection of participants into the study based on characteristics observed after the start of the intervention. The interention started before the follow-up.	Moderate: The interventional group clearly defined, but the definition recorded after the start of the intervention.	Low: No devition from the intended intervention was observed.	Low: No dropouts were reported.	Low: The outcome measure was unlikely to be influenced by knowledge of the intervention received by study participants	Low: The protocol was approved by the institutional review board. The pre-defined outcomes mentioned in the methods section seemed to have been reported.	Serious risk: The study is judged to raise serious risk because one of the domains got this result
Gaffuri et al. (2020) [16]	Low: No confounding is expected.	Low: All participants who would have been eligible for the target trial were included in the study. Furthermore, for each participant, the start of follow-up and the start of intervention coincided.	Low: The interventional group clearly defined at the start of the intervention. classification of intervention status wasn't affected by knowledge of the outcome.	Low: No devition from the intended intervention was observed.	Low: No dropouts were reported.	Low: The outcome measure was unlikely to be influenced by knowledge of the intervention received by study participants	Low: The protocol was approved by the University. The pre-defined outcomes mentioned in the methods section seemed to have been reported.	Low risk: The study is judged to be at low risk of bias for all domains.
Dai et al. (2021) [30]	Low: No confounding is expected.	Serious: Selection of participants into the study based on characteristics observed after the start of the intervention. The interention started before the follow-up.	Moderate: The interventional group clearly defined, but the definition recorded after the start of the intervention.	Low: No devition from the intended intervention was observed.	Low: No dropouts were reported.	Low: The outcome measure was unlikely to be influenced by knowledge of the intervention received by study participants	Low: The protocol was approved by the institutional review board. The pre-defined outcomes mentioned in the methods section seemed to have been reported.	Serious risk: The study is judged to raise serious risk because one of the domains got this result

Effects of Interventions

The main findings of the included studies are given in Table 5. The high clinical heterogeneity among the retrieved studies (variability in the studies' designs, in the studies' outcomes, and the patient's ages) did not allow for conducting a quantitative synthesis of the data in a meta-analysis.

**Table 5:** Overview of the results and conclusions of the included studies ABO-OGS - American Board of Orthodontics - Objective Grading System, LII - Little's irregularity index, PAR - Peer Assessment Rating, CA - clear aligners, FA - fixed appliances

Author (Year)	Results	Conclusion
Baldwin et al. (2008) [26]	During treatment, the average radiographic changes in interdental angle were 21.5° (p<0.0001; n = 10) in the mandible and 16.3° (p<0.0001; n = 19) in the maxilla. On the models, the average changes were 20.8° (p<0.0001; n = 12) in the mandible and 15.9° (p<0.0001; n = 20) in the maxilla No subject completed the initial series of aligners, and only one ultimately completed treatment with aligners.	In premolar extraction patients treated with Invisalign, significant dental tipping occurs (it can be corrected with fixed appliances). There is a trend for greater tipping of mandibular teeth into the extraction space and around the second premolar extraction sites during treatment with aligners.
Li et al. (2015) [27]	Improved total mean scores of the OGS categories after treatment for both groups in terms of alignment, marginal ridges, occlusal relations, overjet,inter-proximal contacts, and root angulation Invisalign® scores were significantly lower than fixed appliance scores for buccolingual inclination and occlusal contacts.	According to the OGS scores, both techniques were successful in the treatment of class I extraction cases. Fixed appliances were superior in buccolingual inclination and occlusal contacts.
Dai et al. (2019) [29]	First molars: Tipped mesially with a difference of 5.86°±3.51°. Translated mesially more than predicted by 2.26±1.56 mm as indicated by mesiobuccal cusp and 2.31±1.67 mm as indicated by distobuccal cusp. The mesiobuccal cusp intruded more than predicted by 0.61±0.89 mm; the distobuccal cusp was stable. Central incisors: Tipped more lingually by 5.16°±5.92°. The retraction was less than predicted by 2.21±1.51 mm and extruded more than predicted by 0.50±1.17 mm.	Retraction of the central incisors and anchorage control of the first molars were not achieved as predicted. Auxiliaries and overcorrection should be considered to help achieve the predicted movement. Achieving the predicted plan is affected by age, attachments, and initial crowding.
Gaffuri et al. (2020) [16]	Improved total mean scores of the OGS categories after treatment for both groups, and there were no significant differences between the two groups. There were no significant differences between the initial and final cephalometric measurements for both intra and inter-group differences.	Both techniques are effective for four premolars extraction cases if accurate diagnosis and appropriate protocols are used.
Dai et al. (2021) [30]	Maxillary first molars: tipped mesially and lingually, whereas they were predicted to tip distally and labially, with significant differences between the predicted and actual changes. For the rotation, there were no significant differences between the changes. Mandibular first molars: there were no significant differences between predicted and actual changes for tipping, but the differences were significant for the rotation with more mesiolingual rotation than predicted. Maxillary canines: No significant changes for the inclination and rotation, whereas the angulation changes were significant with more distal tipping than predicted. Mandibular canines: tipped significantly more than predicted distally and lingually. Rotation was more than predicted mesiobuccally, with no significant differences. Maxillary central incisors: for the angulation and rotation, there were no significant changes between the predicted and achieved changes. Inclination changes were significant towards lingual. Mandibular central incisors: there were significant differences in the inclination and angulation with the teeth tipping toward the distal and lingual. There were no significant changes in the rotation between predicted and actual changes.	Central incisors and canines achieved greater distal tipping, and lingual inclination, and insufficient retraction and intrusion than predicted. First molars showed greater mesial tipping, buccal inclination, rotation, intrusion, mesial displacement, and less constriction than predicted.
Jaber et al. (2022) [28]	No significant differences in the LII for the upper and lower jaws between the two studied groups No significant differences in all studied PAR domains between the two groups. In the CA group, the mean score reduction was 28.39 (±8.51) points, whereas it was 26.39 (±5.76) points in the FA group. In both groups, all the patients were improved. Great improvement was achieved in 88.9% of the patients in the CA group and 91.7% in the FA group. The treatment duration was close in both groups with no significant difference between them.	No significant differences between the CA and FA groups for any of the components of the PAR index after the end of treatment. In-house clear aligners can be effective in the treatment of complex orthodontic cases, including premolars extraction if a suitable teeth movement protocol is used. No difference in the treatment duration were observed with in-house clear aligners and fixed appliances.

Treatment Effectiveness

When occlusal indices were used to evaluate treatment effectiveness, Li et al. found that the total mean scores of the OGS categories improved after treatment in the clear aligners and fixed appliances groups regarding alignment, marginal ridges, occlusal relations, overjet, inter-proximal contacts, and root angulation. In contrast, fixed appliances scores were significantly better than Invisalign® scores for buccolingual inclination and occlusal contacts [27]. Gaffuri et al. found that the clear aligners and fixed appliances techniques improved total mean scores of the OGS categories after treatment, with no significant differences between them [16]. When comparing in-house aligners with conventional fixed appliances, Jaber et al. found no significant differences in the LII for the upper and lower jaws between the two groups. Also, the two groups had no significant differences in all studied PAR domains. The CA group's mean score reduction was 28.39±8.51 points, whereas it was 26.39±5.76 in the FA group. In both groups, 100% of the patients were improved.

In contrast, a great improvement was achieved in 88.9% of the patients in the CA group and 91.7% in the FA group [28]. Baldwin et al. found that the average radiographic changes in interdental angle were 21.5° in the mandible and 16.3° in the maxilla. On the models, the average changes were 20.8° in the mandible and 15.9° in the maxilla. No subject in this study completed the initial series of aligners; only one patient ultimately completed treatment with aligners [26]. When evaluating effectiveness by measuring the tooth movements on superimposed predicted and achieved dental models, Dai et al. found in their first study that the orthodontic treatment with upper first premolars extraction led to a mesial tipping of the first molars with a mean difference of 5.86°±3.51° from the predicted models, mesial translation was also observed for the first molars in a greater extent than the predicted values by a mean of 2.26±1.56 mm and 2.31±1.67 mm when measured at the mesiobuccal cusp and the distobuccal cusp, respectively. For the central incisors, tipping was observed in the actual models more lingually by 5.16°±5.92° than predicted. Also, the retraction of the central incisors was less than predicted by 2.21±1.51 mm, whereas extrusion was more than predicted by 0.50±1.17 mm [29]. In their second study that included orthodontic treatments with upper and lower first premolars extraction, Dai et al. found that the central incisors and canines achieved greater distal tipping and lingual inclination and insufficient retraction and intrusion than predicted. In contrast, the first molars showed greater mesial tipping, buccal inclination, rotation, intrusion, mesial displacement, and less constriction than predicted [30].

Treatment Efficiency

Efficiency was evaluated by calculating the treatment duration. Baldwin et al. found that for premolar extraction cases, clear aligner treatment required more time than treatment with fixed appliances alone [26]. Jaber et al. found that the treatment duration with clear aligners was shorter than that with fixed appliances, with no significant differences between them [28]. In contrast, Li et al. and Gaffuri et al. found that the treatment duration with the fixed appliances was shorter than that with the clear aligners [16,27]. Dai et al. studies found that the first set of clear aligners takes an average of 22.3±4.6 and 21.0±4.2 months in orthodontic treatment with two premolars and four premolars extraction, respectively [29,30].

Discussion

To the best of our knowledge, the present systematic review is the first in the literature to evaluate clear aligners' clinical effectiveness in treating complex orthodontic cases with teeth extraction. This review summarizes the evidence from six trials (involving 283 patients) out of initially identified 1989 studies from the literature, taking into consideration orthodontic treatment using clear aligners in teeth extraction cases. Three trials were judged as having a serious risk of bias [26,29,30], two had some concerns [27,28], and one had a low risk of bias [16]. Participant blinding was the most problematic field. These judges might affect the level of certainty of the achieved results.

Considerable variation in the investigated clinical outcomes was noted among the included studies. When evaluating the achieved occlusal traits after orthodontic treatment, similar levels of effectiveness in achieving acceptable and comparable occlusion were found in both techniques. Upper and lower alignment components were the most improved after orthodontic treatments in both groups. [16,27,28]. Li et al. found that the buccolingual inclination and occlusal contacts were not good as those achieved by the fixed appliances [27]. It is well known that achieving good interocclusal contacts is a key factor in preserving posttreatment stability regardless of the class of malocclusion being treated [31]. Jaber et al. found that the anterioposterior relationships between the upper and lower teeth were lower in the clear aligners groups than the fixed appliances, with no significant differences between them [28].

The differences between the two techniques might be attributed to the precise teeth movements resulting from the accurate positioning of the brackets at the beginning of the treatment and the possibility of repositioning them during the treatment as well as the MBT prescription of the brackets. Using pre-adjusted fixed appliances also allows orthodontists to make precise wire adjustments within 0.5mm to intrude or extrude teeth as necessary and to make fine adjustments with uprighting or rotation springs, interarch elastics, and other auxiliaries [16].

In contrast, extrusion movements with aligners are considered difficult, and teeth occlusal surfaces coverage with the aligners during treatment; both these factors prevented the occlusion's final settling. That is why clear aligners could not produce adequate occlusal contacts compared to the achieved results with fixed appliances. The same problem is found when minor vertical dental movements are required at the end of the orthodontic treatment (i.e., settling) but cannot be achieved due to using vacuum-formed retainers in the retention phase [32]. Moreover, it's hard to guarantee the desired treatment results with clear aligners because success relies on patients' motivation and dependability to complete the treatment, as clear aligners are removable [21].

When aligners' effectiveness was evaluated by tooth movement measurements on superimposed digital dental models, two aspects were evaluated: the anchorage control and teeth retraction by comparing the predicted and achieved movements of the first molars, central incisors, and canines. The first molar anchorage control, represented by mesial tipping and mesial displacement, was not fully achieved as predicted. The amount of maxillary anchorage loss was one-third of the extraction space. In contrast, the mandibular first molar showed better anchorage control than the maxillary first molar, with a smaller achieved amount and a minor difference between achieved and predicted amounts [29,30]. This finding is in accordance with that of fixed orthodontic treatment because the maxillary molars move mesially more easily than the mandibular molars, and the maxillary anterior segment includes larger teeth than the mandibular anterior segment. Retraction of canines and central incisors was less than predicted in the maxilla but the same as predicted in the mandible. Maxillary and mandibular canines achieved notably more distal tipping than predicted, and both maxillary and mandibular central incisors achieved notably more lingual inclination than predicted [29,30]. These findings suggest that tipping movement rather than bodily movement is more likely to occur with canines and central incisors during extraction space closure with clear aligners.

When efficiency was evaluated, different results were found according to the four studies that compared the treatment duration between fixed appliances and clear aligners; three of them found that treatment with clear aligners takes more time than that with fixed appliances [16,26,27], while one study found no differences between the two techniques regarding the treatment duration [28]. The acceleration of orthodontic tooth movement has recently become a critical topic in the orthodontic literature [33-37]. Many physical, biomechanical, and surgical techniques have been suggested to reduce orthodontic treatment time [38-40]. Thus, based on the existing literature about clear aligners, the duration of treatment was shorter with fixed appliances when applied to orthodontic extraction cases. This aspect should be explicitly discussed with patients before choosing the clear aligner treatment modality.

Limitations

There was a lack of large- and high-quality studies investigating the effectiveness of clear aligners in treating complex orthodontic cases with premolars extraction. The number of randomized controlled trials included in this review was small, and cohort studies were included in this systematic review. This might result in bias due to its retrospective design and the bias in selecting participants for these studies. There was incomplete reporting of the potential confounding factors in all studies included in this review, like oral hygiene, compliance, type and number of bonded attachments, number of aligners, rate of refinement aligners needed, or the amount of interproximal reduction (IPR) applied.

## Conclusions

Both clear aligners and fixed appliances are effective in the orthodontic treatment of premolar extraction-based cases. Fixed appliances have the advantage of achieving better buccolingual inclination and occlusal contacts in a shorter treatment duration. Treatment with clear aligners might be associated with differences between predicted and achieved tooth movements. Therefore, the characteristics of these techniques should be considered when making a treatment decision. More well-conducted high-quality studies are needed, with more consideration of the sample size and trial design to study the effectiveness of clear aligners on various types of complex malocclusion cases.
