# Correction: Treatment Effectiveness of Clear Aligners in Correcting Complicated and Severe Malocclusion Cases Compared to Fixed Orthodontic Appliances: A Systematic Review

**DOI:** 10.7759/cureus.c333

**Published:** 2025-10-01

**Authors:** Samer T Jaber, Mohammad Y Hajeer, Kinda Sultan

**Affiliations:** 1 Department of Orthodontics, University of Damascus, Damascus, SYR

This article has been corrected to fix the following typo in the first paragraph of the Results section: "The titles and abstracts of 705 records..." has been corrected to "The titles and abstracts of 703 records...".

